# IL-1R2 as a Precision Therapeutic Target in Sepsis: Molecular Insights into Immune Regulation

**DOI:** 10.3390/cimb47060429

**Published:** 2025-06-06

**Authors:** Kirtan Dave, Cristian R. Munteanu

**Affiliations:** 1 Parul Institute of Applied Sciences, Parul University, Vadodara 391760, Gujarat, India; kirtandave11@gmail.com; 2 Bioinformatics Laboratory, Research & Development Cell, Parul University, Vadodara 391760, Gujarat, India; 3 RNASA, Computer Science Faculty, University of A Coruña, 15071 A Coruña, Spain

**Keywords:** sepsis, immune response, inflammation, IL-1R2, NFκB signaling, biomarkers, therapeutic targets

## Abstract

Sepsis is a life-threatening condition characterized by systemic inflammation and organ dysfunction, with a complex and not yet fully elucidated molecular basis. Central to its pathogenesis is a dysregulated immune response. In this study, we performed a comprehensive multi-omics analysis on transcriptomic datasets retrieved from the GEO database, including samples from sepsis patients (*n* = 23) and healthy controls (*n* = 27). and identified a pivotal role of Interleukin-1 receptor 2 (IL-1R2) in modulating inflammatory responses in sepsis. Transcriptomic integration revealed activation of critical signaling pathways, including NFκB/NLRP3, associated with sepsis-induced immune dysregulation. We identified a pivotal role of Interleukin-1 receptor 2 (IL-1R2) in modulating inflammatory responses in sepsis, with IL-1R2 showing a 2.1-fold upregulation in septic patients. Transcriptomic integration revealed the activation of 42 significantly enriched signaling pathways, with 26 upregulated and 26 downregulated pathways. Notably, the NFκB/NLRP3 signaling axis emerged as a central hub of immune dysregulation. Gene Ontology (GO) enrichment analysis highlighted “neutrophil activation involved in immune response” as the top biological process. Our findings suggest that IL-1R2 functions as a key immunoregulatory molecule and represents a promising therapeutic target. Moreover, we observed distinct patterns of oxidative stress regulation and immune cell activation, with potential biomarkers correlating with disease severity. These insights not only enhance the molecular understanding of sepsis but also point toward novel precision therapeutic strategies focused on modulating inflammation to improve patient outcomes.

## 1. Introduction

One of the life-threatening conditions characterized by erratically regulated systemic inflammation and immune responses to infection is sepsis, often leading to organ failure and high mortality rates [1]. Despite advances in understanding its pathogenesis, the molecular mechanisms triggering sepsis and its regulation remain inadequately understood, necessitating the identification of novel biomarkers and therapeutic targets to improve clinical outcomes [2].

**Figure 1:**
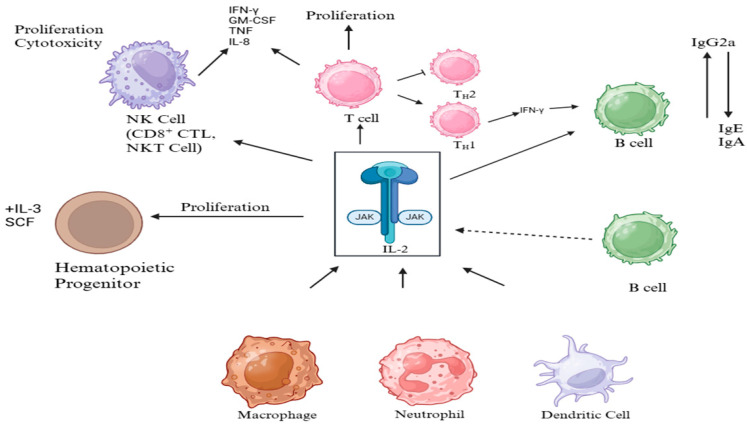
IL-2 mediated immune response pathway: Interactions of IL-2 with various immune cells, highlighting proliferation, differentiation, and cytokine production.

Central to the pathogenesis of sepsis are cytokine-mediated signaling cascades (Figure 1). For instance, the IL-6–driven JAK/STAT3 pathway is known to mediate immune activation, inflammatory amplification, and cellular survival mechanisms [3,4]. Members of the interleukin-1 receptor family, including IL-1 receptor type 1 (IL-1R1), IL-18 receptor 1 (IL-18R1), and IL-1 receptor type 2 (IL-1R2), play critical roles in regulating these inflammatory processes. While IL-1R1 and IL-18R1 initiate pro-inflammatory signaling via NF-κB and MAPK pathways, promoting IL-6 production, IL-1R2 functions as a decoy receptor. By binding IL-1β without signaling, IL-1R2 serves as a negative regulator, attenuating excessive inflammatory responses [5,6].

The balance between pro- and anti-inflammatory signaling is crucial for immune homeostasis, and recent studies have highlighted the significance of regulatory mechanisms that suppress uncontrolled inflammation in sepsis [7]. For example, ALKBH8, a human AlkB homolog, has been reported to mitigate inflammation by limiting oxidative stress and inhibiting the NF-κB/NLRP3 axis, thereby reducing downstream cytokine release [8]. Furthermore, clinical investigations into immunomodulatory agents, such as clarithromycin, have demonstrated their potential to improve survival outcomes in septic patients by modulating transcriptional responses and dampening cytokine storms [9].

Given the complexity of immune regulation in sepsis, an integrative transcriptomic approach can provide comprehensive insights into the molecular networks involved. Such methods allow the identification of key regulators, including decoy receptors like IL-1R2, and help contextualize their role within broader inflammatory and immunosuppressive pathways.

In this study, the objective is to investigate the regulatory role of IL-1R2 as a potential biomarker for sepsis and its connection to key inflammatory pathways, including the IL-6/JAK/STAT3 axis. We further explore the interplay between IL-1R2 and immune-suppressive mechanisms such as ALKBH8-mediated NF-κB/NLRP3 signaling suppression. In addition, we examine the contributions of the p53 signaling pathway, which regulates cellular stress responses and apoptosis, and the IL-2/STAT5 signaling axis, known for its role in T cell survival and immune modulation during infection. We also evaluate the clinical relevance of immunomodulatory therapy, such as clarithromycin, in altering these pathways to improve outcomes in septic patients. By integrating transcriptomic profiling with mechanistic and therapeutic perspectives, this study aims to advance our understanding of sepsis pathogenesis and identify innovative strategies for targeted immunomodulation.

## 2. Materials and Methods

### 2.1. Data Collection

The National Center for Biotechnology Information’s (NCBI) Gene Expression Omnibus (GEO) database was used to acquire datasets of gene expression. RNA-seq datasets were retrieved to for identification of genes common to both sepsis and inflammation. The search was conducted using keywords like “sepsis”, “blood”, and “leukocyte”. The Fastq files were downloaded from GEO accession IDs GSE196117 [10] (*n* = 40) = 6 control + 32 sepsis and GSE211210 [11] (*n* = 10) = 5 control + 5 sepsis. These datasets comprised RNA-seq expression data from sepsis, control, treated and placebo. A total of 50 samples from studies on sepsis patients were analyzed, and a comparison between infected samples and control samples was conducted. A comprehensive summary of the RNA-seq data utilized in this study is covered in Table S1, covering placebo and sepsis samples from numerous studies. It includes detailed information such as GEO IDs, sample titles, run accessions, and specific study classifications.

### 2.2. Sequence Alignment and Transcriptome Assembly

**Figure 2:**
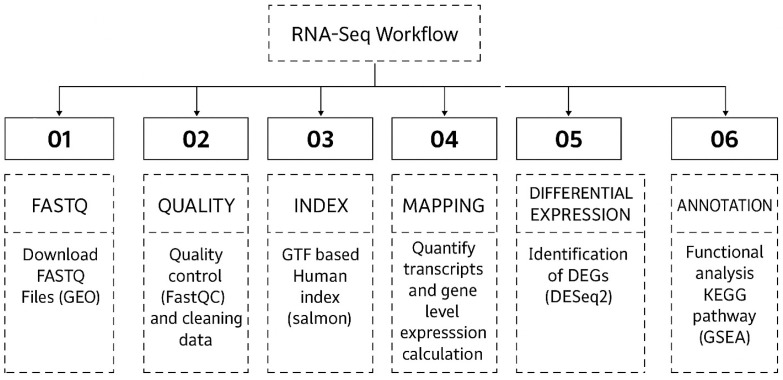
Schematic overview of the Bioinformatics analysis workflow.

A uniform RNA-seq analysis pipeline was applied to the 54 downloaded RNA-seq runs. RNA-seq runs were downloaded and analyzed for sequencing errors using FastQC (http://www.bioinformatics.babraham.ac.uk/projects/fastqc) (accessed on 30 May 2025) in initial quality control. Second, Fastp (version 0.23.0) was used to reduce the transcript abundance from individual samples as part of quality control [12]. In particular, taking into account the presence of infected and mock cells from different investigations, we employed quality-controlled RNA-seq runs with parameters of phred scores of 15 and a minimum length requirement of 20 [13]. Next, we determined the expression levels in each sample using transcripts per million (TPM). After compiling all of the samples’ quantitative data into an expression matrix, we normalized the expression levels across samples using a trimmed mean of M values (TMM) [14]. To evaluate the degree of tissue specificity in gene expression, we calculated the τ value for each gene [15]. R Studio version 3.4 (Bioconductor Package 3.18) was utilized for the analysis. The “enrichplot” package (https://bioconductor.org/packages/release/bioc/html/enrichplot.html, accessed on 30 May 2025) was used to create the bar plot displaying the top 20 significantly enriched pathways [16], and the “GSVA” R package (https://bioconductor.org/packages/release/bioc/html/GSVA.html, accessed on 30 May 2025) was used to create the heatmap [17]. A bioinformatics pipeline schematic is included in the Bioinformatics Workflow in Figure 2.

## 3. Results

### 3.1. Determination of Common Differentially Expressed Genes

**Figure 3:**
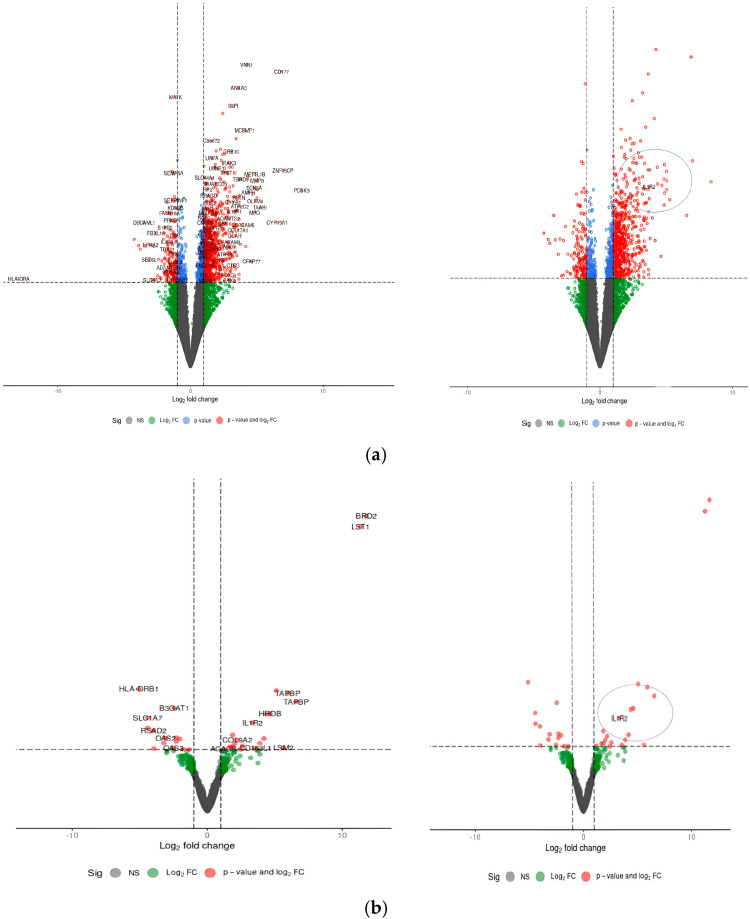
(**a**,**b**) GSE196117 (*n*  =  40) and GSE211210 (*n*  =  10). The red points show up-regulated genes (log2 FC  ≥  0.5 and adjusted *p*-value  <  0.05). The *x*-axis shows the log2 fold change, indicating the level of gene expression differences, while the *y*-axis indicates the statistical significance (−log10 *p*-value). The IL1R2 gene is significantly upregulated, and is marked in red and surrounded by a circle.

The differences in gene expression between sepsis and control samples were analyzed to identify common genes with variation in their expression. The genes that showed significant expression in transcript clusters are compared, illustrated in Table S2. In R Studio, DESeq2 [18] was used for the analysis, and the adjusted *p*-value was less than 0.05. Following the mapping of genomic data from PBMCs of patients with severe sepsis and PBMCs from healthy patients, heatmaps of genes with differential expression showed clustering based on transcriptome profiles and states (infected and sepsis). The analysis of GSE196117 (*n* = 40) and GSE211210 (*n* = 10) demonstrates a significant quantity of genes exhibiting modified expression levels under the given circumstances. The study GSE196117 identified 2315 genes that were upregulated, and 932 genes were found to be upregulated in GSE251849. Both studies met the criterion of having an adjusted *p*-value of less than 0.05 and log-fold change thresholds. To see changes in expression and their significance throughout the full gene set, Figure 3 shows a volcano plot of statistical significance (FDR or *p*-value) vs. log fold change. In two dimensions, every gene is depicted as a point. A summary of the commonly upregulated DEGs is shown in Table 1.

### 3.2. Analysis of the Pathways Associated with Significantly Dysregulated Transcriptome Data of Both the Datasets

**Figure 4:**
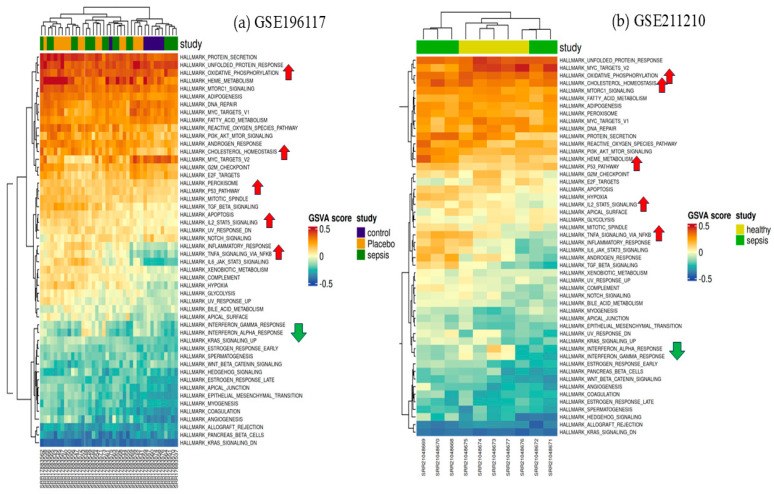
Heat map of the top differentially expressed genes based on GSE196117 (*n* = 40) and GSE211210 (*n* = 10). The color intensity suggests the higher to lower expression pathway and pathway enrichment analysis.

Figure 4 predicts the impact of dysregulated genes across various sample categories in both datasets. The P53 process, IL2/STAT5 signaling, cholesterol homeostasis, and oxidative phosphorylation demonstrate a significant correlation between transcriptomic dysregulation and its impact on this essential metabolic process. The deregulation of these pathways contributes to immune regulatory failure; this interaction may affect the overexpression of IL1R2, a recognized decoy receptor with anti-inflammatory functions in sepsis, underscoring the immunological-metabolic reprogramming involved in the course of severe sepsis. The pathways of interferon gamma response and interferon alpha response were shown to be downregulated in samples with increased IL1R2 expression. The inverse association indicates that IL1R2, functioning as a decoy for interleukin-1 (IL-1), may assist in regulating the broader network of cytokine signals, encompassing interferon pathways.

### 3.3. Gene Ontologies and Enrichment

**Figure 5:**
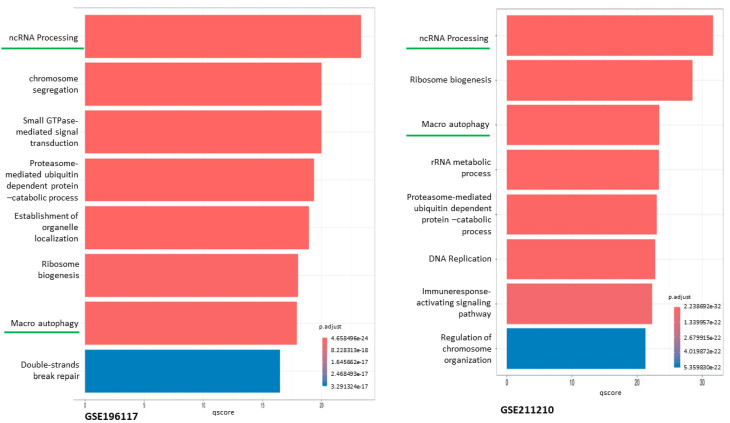
Gene Ontology (GO) enrichment analysis indicates that differentially expressed genes (DEGs) are primarily enriched in biological processes, including non-coding RNA processing and proteasome-mediated protein degradation, both of which are associated with sepsis-related inflammatory activity.

The clusterProfiler package provides the enrichGO functions for ORA and GSEA using GO, along with genome-wide annotation packages [19]. Gene Ontology (GO) [20] analyses (Figure 5) of the top DEGs revealed significant enrichment in immune system processes, inflammatory responses, and IL2 signaling. IL1R2 was identified in several of these pathways, particularly those linked to cytokine-cytokine receptor interaction, regulation of inflammatory responses, and negative regulation of IL-1 signaling.

GO enrichment [21] also indicated involvement in ncRNA processing and proteasome-mediated protein catabolism, which are associated with immune suppression in critical illness. These pathways collectively underscore the immune dysregulation seen in sepsis, suggesting that IL1R2 plays a central regulatory role [22].

### 3.4. Network Analysis and Key Inflammatory Genes

**Figure 6:**
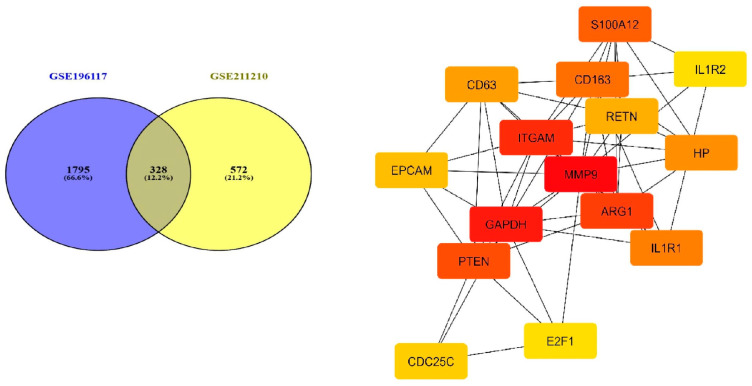
Venn plot of differentially expressed genes between the sepsis- group and control group. (Software: R (4.5) version, R packet: VennDiagram (1.7.3). URL: https://cran.rstudio.com/web/packages/VennDiagram/index.html, accessed on 30 May 2025) and PPI module plot of immune-related key genes. PPI analysis showed widely connection of immune-related genes, among which SA00A12, IL1R2, IL1R1 MMP9 ARG1 are located in the center of the network.

In two investigations, a comparative examination of gene expression between sepsis and healthy patient groups demonstrated that 328 genes were upregulated, as indicated in Supplementary Table S3. Multiple genes exhibited increased levels consistently across groups, as seen in the Venn diagram in [23]. A total of 328 proteins associated with the common differentially expressed genes were identified and incorporated into the protein–protein interaction in network shows in Figure 6. To investigate their molecular interactions, we established a protein–protein interaction (PPI) network utilizing the STRING database [24] (confidence score > 0.7), which unveiled a complex interaction landscape. The resultant PPI network was imported into Cytoscape 3.9.1 [25], where the topological analysis plugin CytoHubba [26] was utilized to prioritize hub genes according to various centrality approaches. This analysis identified the top 15 hub genes exhibiting the highest connectivity, underscoring their essential roles in preserving network stability and potentially influencing sepsis pathophysiology. The findings indicated that these genes were significantly engaged in the network regulated by IL1R2 signaling and were notably associated with infected cells. Network topology analysis indicated that numerous hub genes belong to a closely interconnected module governed by IL1R2 signaling, a mechanism increasingly associated with immunological dysregulation in sepsis.

## 4. Discussion

The study was performed to find evidence to establish the role of IL1R2 in controlling the immune response during sepsis. As a bait receptor for IL-1β, IL-1R2 has a two-fold function in the pathophysiology of sepsis. It works by reducing hyperinflammation in the early stages of the illness and, paradoxically, promoting immune suppression in the later stages [27,28]. This dual role reflects the intricate immunological dysregulation that characterizes sepsis, in which pro-inflammatory and immunosuppressive states coexist and influence disease progression and patient outcomes. Sepsis patients exhibited a distinct pattern, with decreased sIL-1R1 plasma concentrations but markedly increased levels of IL-1R2 and IL-1R1 [29].

Further, it emphasized the central role of NF-κB/NLRP3 signaling pathway is in sepsis and how IL-1R2-mediated inhibition of this axis leads to the decrease in recruitment and activation of effector cells of immune system. In particular, the inhibition of T cells, Th17 cells, and NK cells impairs and diminishes the immune system’s ability to clear infections and contributes to immunoparalysis. This immunoparalysis is a defining feature of sepsis. This dysfunction is increased by the downregulation of crucial immune pathways, such as Th1 and Th2 differentiation [30], antigen presentation, and NK cell-mediated cytotoxicity. When combined, these dysregulated mechanisms produce a feedback loop that amplifies immunological suppression and worsens patient outcomes [31,32].

Pathway enrichment analysis further highlighted the importance of IL-1R2 in regulating immune and inflammatory responses. Notably, the study reveals a functional interaction between IL-1R2 and the IL-6/JAK/STAT3 axis [33], as well as potential crosstalk with the IL-2/STAT5 signaling pathway, which plays a vital role in T cell proliferation, differentiation, and survival [34]. Disruption of IL-2/STAT5 signaling in sepsis contributes to lymphopenia and T cell exhaustion, reinforcing immunosuppressive conditions [35,36]. These findings underscore the interconnected nature of cytokine signaling cascades in shaping the immune landscape during sepsis [37].

In addition, the TNF-α/NF-κB pathway, a major inflammatory driver in sepsis, is modulated by IL-1R2 activity. Downregulation of this axis through IL-1R2 may reduce excessive inflammation, but prolonged inhibition could impair host defense, especially against secondary infections. This supports a therapeutic model where timing and context of pathway modulation are critical for successful outcomes [38].

Interestingly, emerging evidence from multi-omics analysis links sepsis pathophysiology to cholesterol homeostasis. IL-1R2 expression appears to correlate with altered expression of genes involved in cholesterol biosynthesis and transport, suggesting that metabolic dysregulation plays a role in immunosuppression. Given that cholesterol-rich membrane microdomains are essential for immune receptor signaling and antigen presentation, disturbances in cholesterol balance could further impair immune cell function [39].

Furthermore, the study implicates the p53 signaling pathway in the immune-metabolic stress response during sepsis. p53, a well-known regulator of apoptosis and cellular stress responses, may be activated in immune cells during systemic inflammation, leading to increased cell death and contributing to lymphocyte depletion. IL-1R2’s potential influence on this pathway highlights its role not only in immune regulation but also in cellular fate decisions under septic stress [40].

Finally, the study identifies dysregulation in oxidative phosphorylation (OXPHOS)—a critical metabolic process for ATP production—in patients with sepsis. IL-1R2 expression was associated with suppression of mitochondrial respiration genes, consistent with the metabolic reprogramming observed in septic immune cells. Impaired OXPHOS may compromise immune cell energy supply, limiting their effector functions and contributing to immunoparalysis [41,42].

With a multi-omics approach that combines many diverse data sources, such as RNA sequencing and protein–protein interaction networks, scientists have been able to learn more about the molecular processes that underlie sepsis. Recent advancements in this approach have considerably improved identification of key biomarkers, pathways, and potential pharmaceutical targets. This comprehensive study has improved our understanding of the molecular mechanisms causing sepsis, emphasized the importance of a balanced immune response, and revealed the progression of the disease. Through the integration of clinical data and transcriptome insights, the study identifies prospective treatment approaches targeted at immune function restoration. Notably, there is potential for improving patient outcomes by focusing on important signaling molecules such NF-κB and the interleukin-1 receptor type 2 (IL-1R2). In addition to improving our knowledge of sepsis, this integrated approach opens the door to more personalized treatment options that may have a major influence on survival and recovery rates.

## 5. Conclusions

In conclusion, this study helps us advance our understanding of sepsis pathophysiology by highlighting IL-1R2 as an important regulator of inflammation and immune suppression. The findings suggest that IL-1R2 could serve as both a prognostic biomarker and a potential pharmaceutical target. The aim of future research should be to create IL-1R2-focused treatments, learn more about the complex molecular networks involved in sepsis, and investigate novel approaches to help individuals with the illness regain their immune systems.

## Figures and Tables

**Table 1:** Gene Expression Omnibus (GEO) accession information for samples.

GEO Accession	GSE196117	GSE211210
Sample No	*n* = 40	*n* = 10
Title of study	Transcriptional profiling of whole blood leukocytes obtained from critically ill patients with sepsis treated with clarithromycin, or not, and healthy participants	ALKBH8 suppresses oxidative stress and ameliorates inflammation via blocking NFκB/NLRP3 axis in Sepsis
Differential Genes with adjusted *p*-value < 0.05	2315 genes Upregulated	932 genes Upregulated
	1728 genes down regulated	707 genes down regulated

## Data Availability

The datasets analyzed during the current study are publicly available in the NCBI Gene Expression Omnibus (GEO), a repository recommended by MDPI. The following GEO accession numbers were used: GSE196117: https://www.ncbi.nlm.nih.gov/geo/query/acc.cgi?acc=GSE196117 (accessed on 30 May 2025). GSE167363: https://www.ncbi.nlm.nih.gov/geo/query/acc.cgi?acc=GSE211210 (accessed on 30 May 2025).

## Supplementary Materials

The following supporting information can be downloaded at https://www.mdpi.com/article/10.3390/cimb47060429/s1.

## Abbreviations

NLRP3	NLR Family Pyrin Domain Containing 3
ROS	Reactive Oxygen Species
OxS	Oxidative Stress
Th17	T-helper 17 Cells
PBMCs	Peripheral Blood Mononuclear Cells
