# Supplementary material for: IL-1R2 as a Precision Therapeutic Target in Sepsis: Molecular Insights into Immune Regulation
Source: Curr Issues Mol Biol. 2025 Jun 6;47(6):429. doi: 10.3390/cimb47060429 (PMC12192091; doi:10.3390/cimb47060429)
Supplement: Supplementary file 1 [file cimb-47-00429-s001.zip › Table S1.pdf]

1)GSE211210	Library Name	condition	source_name
	GSM5860293	sepsis	whole blood
https://www.ncbi.nlm.nih.gov/geo/query/acc.cgi?acc=GSE196117	GSM5860292	sepsis	whole blood
	GSM5860291	sepsis	whole blood
	GSM5860290	sepsis	whole blood
	GSM5860289	sepsis	whole blood
	GSM5860288	sepsis	whole blood
	GSM5860287	sepsis	whole blood
	GSM5860286	sepsis	whole blood
	GSM5860285	sepsis	whole blood
	GSM5860284	sepsis	whole blood
	GSM5860283	sepsis	whole blood
	GSM5860282	sepsis	whole blood
	GSM5860281	sepsis	whole blood
	GSM5860280	sepsis	whole blood
	GSM5860279	sepsis	whole blood
	GSM5860278	sepsis	whole blood
	GSM5860277	sepsis	whole blood
	GSM5860276	sepsis	whole blood
	GSM5860275	sepsis	whole blood
	GSM5860274	sepsis	whole blood
	GSM5860273	control	whole blood
	GSM5860272	control	whole blood
	GSM5860271	control	whole blood

	GSM5860270	control	whole blood
	GSM5860306	sepsis	whole blood
	GSM5860305	sepsis	whole blood
	GSM5860304	sepsis	whole blood
	GSM5860303	sepsis	whole blood
	GSM5860302	sepsis	whole blood
	GSM5860301	sepsis	whole blood
	GSM5860300	sepsis	whole blood
	GSM5860299	sepsis	whole blood
	GSM5860298	sepsis	whole blood
	GSM5860297	sepsis	whole blood
	GSM5860296	sepsis	whole blood
	GSM5860295	sepsis	whole blood
	GSM5860294	sepsis	whole blood
	GSM5860269	control	whole blood
	GSM5860268	control	whole blood
	GSM5860267	control	whole blood
2) GSE196117	Library Name	condition	source_name
https://www.ncbi.nlm.nih.gov/geo/query/acc.cgi?acc=GSE196117	GSM6456152	patients with acute sepsis	blood
	GSM6456151	patients with acute sepsis	blood
	GSM6456150	patients with acute sepsis	blood

	GSM6456149	patients with acute sepsis	blood
	GSM6456148	patients with acute sepsis	blood
	GSM6456147	health donors	blood
	GSM6456146	health donors	blood
	GSM6456145	health donors	blood
	GSM6456144	health donors	blood
	GSM6456143	health donors	blood
